# Auditory object segmentation amplifies prediction error signals in a complex MMN paradigm

**DOI:** 10.3389/fnins.2025.1695952

**Published:** 2025-12-05

**Authors:** Fran López-Caballero, Dylan Seebold, Hayley Rhorer, Lauren Fowler, Jack Kavanagh, Sophia Yi, Alfredo L. Sklar, Brian A. Coffman, Dean F. Salisbury

**Affiliations:** Clinical Neurophysiology Research Laboratory, Western Psychiatric Hospital, University of Pittsburgh School of Medicine, Pittsburgh, PA, United States

**Keywords:** complex mismatch negativity, auditory scene analysis, auditory segmentation, MEG, source solutions

## Abstract

Auditory deviance detection depends on the segmentation of sound sequences into perceptually meaningful units, which provide the basis for predictive models. Complex mismatch negativity (MMN) paradigms, including dual-rule designs, place higher demands on these processes because regularities are defined by relationships across tones rather than by single features. However, it remains unclear how facilitation of auditory segmentation influences MMN. To address this, we compared MMN elicited during a dual-rule paradigm under continuous versus temporally segmented (750 ms gap) conditions in thirty-two participants using simultaneous EEG and MEG recordings. Two 50-ms binaural pure tones were presented in pairs. Tone A was a 1 kHz stimulus biased to the right ear, and Tone B was a 1.2 kHz stimulus biased to the left ear. Within each pair, tones followed with a 330 ms Stimulus Onset Asynchrony (SOA), with standards consisting of A-B pairs (85.6%) and deviants of A-A pairs (14.3%). Two conditions were used: a No-Gap condition, where pairs followed continuously with a 330 ms SOA, and a Gap condition, where pairs were separated by a longer 750 ms SOA. Results at EEG FCz revealed dual-rule MMN amplitudes (130–230 ms) were not significantly different between No-Gap and Gap conditions (t_(29)_ = 0.93; *p* = 0.36; *d* = 0.17). Conversely, MEG source-localized responses in bilateral primary auditory cortex (A1) revealed larger MMN responses in the Gap condition relative to No-Gap (*F*_(1,31)_ = 26.07, *p* < 0.001, $\eta_{p}^{2}$ = 0.457). This suggests that temporal segmentation facilitates the grouping of tone pairs into perceptual objects, allowing the auditory system to form predictions not only at the level of individual tones, but also at the level of the tone pair as a perceptual unit. Deviants in this context violate both low-level features (pitch and location) and the higher-order pair structure, producing larger prediction error signals. These results provide insight into how the auditory system integrates local and higher-order regularities, offering a mechanistic link between auditory scene analysis and hierarchical predictive coding in complex auditory environments.

## 1 Introduction

Auditory perception relies on the brain’s ability to organize continuous acoustic input into meaningful perceptual units. This process, known as auditory scene analysis (ASA; Bregman and McAdams, 1994; Szabó et al., 2016), allows listeners to identify distinct sound sources, integrate acoustic elements that belong together, and segment sequences into auditory objects (Bizley and Cohen, 2013). ASA involves at least two complementary operations. Segregation refers to the separation of simultaneously occurring sounds into distinct perceptual streams, such as distinguishing a speaker’s voice from background noise. Segmentation, by contrast, refers to the grouping of sequential acoustic events into coherent auditory objects, such as parsing words in a sentence or tones in a melody (Bregman and McAdams, 1994). Without segmentation, the auditory system would be exposed to a continuous stream of unrelated sounds, with no structure for prediction and interpretation.

Electrophysiological evidence has identified reliable markers of auditory object segmentation. Event-related potential (ERP) studies show that tones at the beginning and end of a sequence evoke enhanced N2 amplitudes, interpreted as a form of “edge detection” that signals the initiation and closure of auditory objects (Chait et al., 2008). In addition, sustained ERP responses have been observed throughout the duration of grouped tone patterns, returning to baseline at group offset. This sustained response, named the auditory segmentation potential (ASP), has been proposed as a neural correlate of object-level grouping, acting as a binding signal that holds elements together in perceptual memory (Coffman et al., 2016, 2018). Similar sustained responses have been described in relation to long-duration tones or sequences with strong temporal predictability (Auksztulewicz et al., 2017; Barascud et al., 2016; Picton et al., 1978; Southwell et al., 2017). Importantly, segmentation processes appear to be largely automatic, as the auditory cortex can extract temporal structure and form perceptual groups without the need for directed attention (Näätänen et al., 2001).

Segmentation mechanisms are closely linked to auditory deviance detection, typically measured through the mismatch negativity (MMN) response. MMN is an ERP component measured with EEG or MEG and elicited when an infrequent stimulus violates an established regularity in an ongoing sequence (Näätänen et al., 1978, 2007). In the auditory domain, classical oddball paradigms use simple deviations in features such as pitch, duration, or intensity (e.g., Murphy et al., 2020; Todd et al., 2014). However, interpretations of these simple MMN paradigms are biased by stimulus-specific adaptation (SSA): responses to repeated standards are suppressed, whereas deviants, being novel, are released from adaptation (May and Tiitinen, 2010). Thus, the MMN reflects a mixture of true deviance detection and release from adaptation (Carbajal and Malmierca, 2018). To minimize this confound, researchers have employed complex MMN paradigms in which deviants violate higher-order rules rather than single features. In these tasks, deviants may disrupt a pattern of relationships between tones, such as sequence length (Rudolph et al., 2014; Salisbury, 2012) or pitch alternations (Saarinen et al., 1992; Van Zuijen et al., 2004). In such designs, deviants and standards are equally adapted, ensuring that any negativity reflects prediction error to a broken rule rather than a new physical stimulus. Crucially, detecting these complex deviants depends on the auditory system’s capacity for segmentation and object formation. The brain must first organize the ongoing sequence into structured units before comparing incoming input against expectations derived from these units (Hikita et al., 2020; Sussman, 2005). Complex MMN paradigms may place especially high demands on this segmentation process, since regularities are defined by relationships across tones rather than single features.

A few studies have suggested a close relationship between auditory segmentation processes and MMN, even though this link has not been directly assessed. Altering the temporal arrangement of tones, which hinders perceptual segmentation, can reduce or abolish the MMN (Atienza et al., 2003; Sussman and Gumenyuk, 2005). This is consistent with the idea that without segmentation into coherent objects, there is no stable template against which to detect deviations. Similarly, MMN amplitudes are enhanced when perceptual grouping is facilitated, for instance when tones are arranged so they are more easily organized into separate streams (Smith and Joshi, 2014). From a theoretical perspective, MMN can be understood within the predictive coding framework. In this account, the auditory system continuously generates predictions based on regularities in the input and updates them when violations occur (Winkler, 2007). Winkler (2007) argued that the predictive models underlying MMN encode temporally structured relationships between sounds. Importantly, these predictive models provide the basis for temporal grouping, a prerequisite for forming auditory objects. Thus, MMN should not be viewed simply as a marker of deviance but rather as a reflection of the ongoing process of building and updating internal models that enable segmentation and object formation.

While both empirical evidence and conceptual models point to a connection between segmentation and deviance detection, to the best of our knowledge, no studies have directly examined how segmentation-related neural activity may modulate MMN amplitude in complex MMN designs.

The present study addresses this issue by directly manipulating temporal context to facilitate segmentation in a complex MMN paradigm. Participants listened to alternating pairs of tones differing in pitch and lateralization. Infrequent deviants violated both dimensions simultaneously, requiring detection of a higher-order regularity. To manipulate segmentation, we compared a No-Gap condition, where pairs followed continuously, with a Gap condition, where pairs were separated by a 750 ms interval. We hypothesized that segmentation into perceptual units would enhance MMN responses by allowing the auditory system to generate predictions not only at the level of individual tones but also at the level of more distinctly perceived auditory objects (pairs). By recording both EEG and MEG, we sought to characterize how segmentation modulates MMN at the scalp and localize the neural generators of this effect within the auditory cortex. This approach offers novel insight into how the brain integrates segmentation and deviance detection, providing a link between auditory scene analysis and predictive coding models of MMN.

## 2 Materials and methods

### 2.1 Participants

Thirty-two healthy participants (14 males) completed the study, ranging in age from 18 to 37 years (mean age: 24.62 years, SD: 4.68). Participants were excluded if they reported a history of concussion or head injury with lasting effects, alcohol or drug dependence within the past 5 years, or any neurological or psychiatric comorbidity. Prior to the experiment, all participants underwent pure-tone audiometry testing (1000–4,000 Hz) to confirm average hearing thresholds below 30 dBnHL in each ear and interaural differences smaller than 15 dB. The study protocol was approved by the University of Pittsburgh Institutional Review Board and adhered to the Declaration of Helsinki. Written informed consent was obtained from all individuals, who received monetary compensation for their participation.

### 2.2 Stimuli and procedure

**Figure 1:**
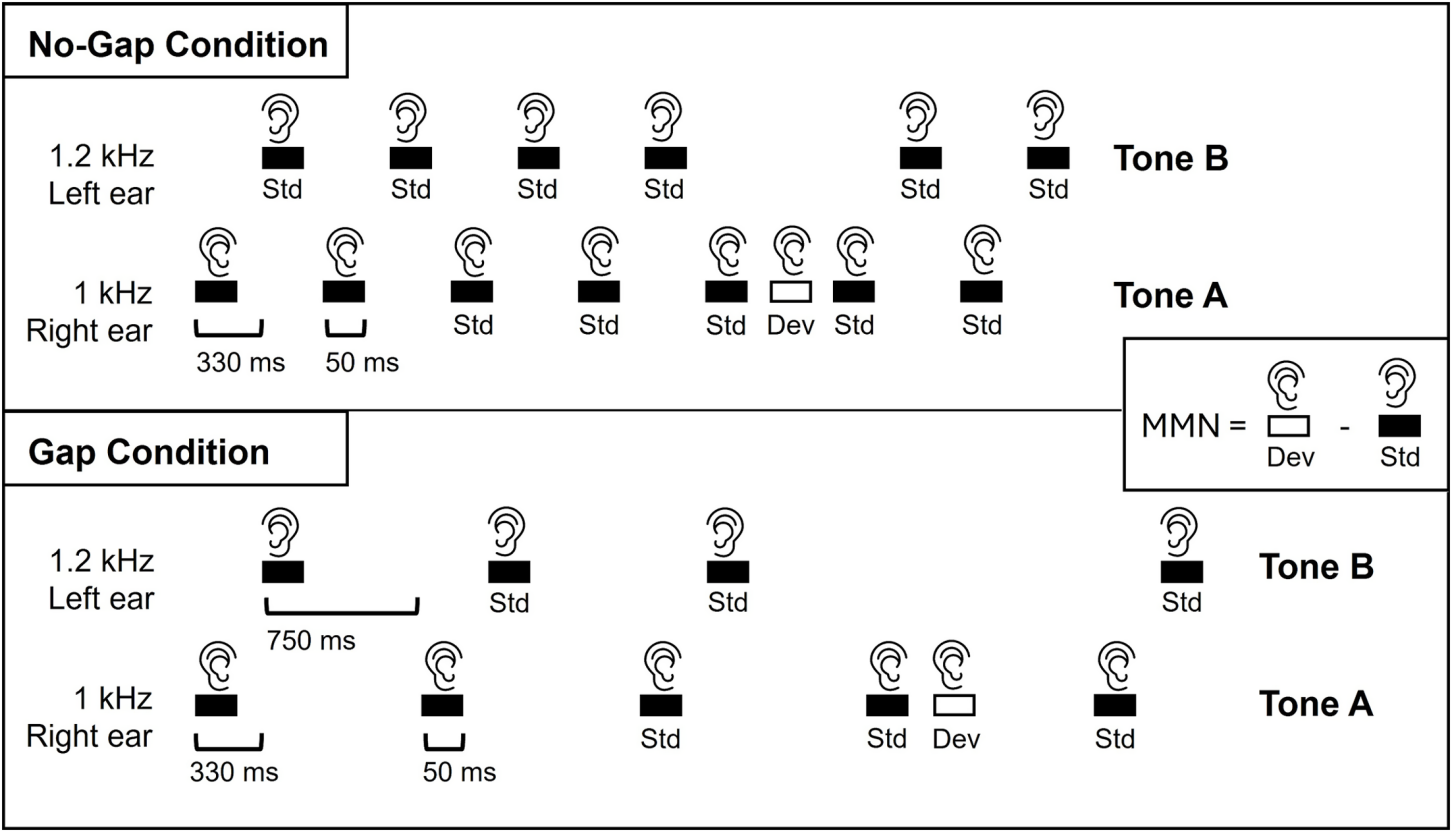
Example of the auditory stimulation sequence. Stimuli were 50-ms binaural tones presented in alternating pairs. Tone A (1 kHz) was biased to the right ear (80 dB right, 65 dB left), and Tone B (1.2 kHz) was biased to the left ear (80 dB left, 65 dB right). Standard pairs (A-B, 85.6%) were presented along with occasional deviant pairs (A-A, 14.3%). Two presentation modes were used: a No-Gap condition, where pairs followed continuously with a 330 ms SOA, and a Gap condition, where pairs were separated by 750 ms. Dual-rule MMN was derived by subtracting the response to standards (Tone B in A-B pairs) from the response to deviants (repeated Tone A in A-A pairs).

Stimuli consisted of two binaural pure tones differing in frequency and lateralization (Figure 1). Tone A was a 1 kHz, 50-ms tone (5-ms rise/fall) biased toward the right ear (80 dB right, 65 dB left), whereas Tone B was a 1.2 kHz, 50-ms tone biased toward the left ear (80 dB left, 65 dB right). Tones were presented with a SOA of 330 ms. Standard sequences followed the alternating pattern A-B (right-low, left-high), while deviants occurred when Tone A was repeated in place of Tone B, producing an A-A pair (right-low, right-low). Thus, the deviant violated expectations in both pitch and lateralization. Two timing conditions were employed: in the No-Gap condition, pairs were presented continuously with a 330 ms SOA, whereas in the Gap condition pairs were separated by 750 ms. A within-pair SOA of 330 ms (i.e., ~300 ms range) is sufficient to elicit reliable MMN responses (Näätänen et al., 2007) and has been shown to produce robust and replicable effects in previous auditory oddball and regularity paradigms (Escera et al., 1998; Jääskeläinen et al., 1999). In addition, we selected a 750 ms SOA between tone pairs in the Gap condition to provide a clear temporal boundary, minimizing overlap between ERPs to successive pairs while promoting perceptual segmentation into discrete tone-pair objects. This interval is long enough to allow a clean separation of responses but not so long as to unnecessarily prolong the task or weaken the predictive context needed for MMN generation. For each condition, 700 tone pairs were delivered (600 standards, 100 deviants; 14.3%), with at least two standard pairs separating any deviant occurrences. All stimuli were created using Ace of Wav (Polyhedric Software) and delivered through ER-3A insert earphones (Etymotic Research, Inc., Elk Grove Village, IL, USA) using Presentation® software (Version 25.0).

During the recordings, participants sat in the MEG/EEG scanner in a magnetically shielded room while passively listening to the tone sequence and while MEG & EEG were recorded. They were instructed to watch a silent movie without subtitles and ignore the sounds as best as possible.

### 2.3 MRI acquisition and processing

For each participant, structural and functional MRI data were collected to support MEG source modeling. T1-weighted (T1w) anatomical images were acquired on a Siemens 3 T MAGNETOM Prisma scanner using a multi-echo 3D MPRAGE sequence (TR/TE/TI = 2400/2.22/1000 ms, flip angle = 7°, FOV = 256 × 240 mm, 0.8 mm isotropic resolution, 208 slices, GRAPPA factor = 2). High-resolution T2-weighted (T2w) images were obtained using a T2-SPACE sequence (TR = 3,200 ms, TE = 563 ms, FOV = 256 × 240 mm, 0.8 mm isotropic, 208 slices), together with a standard field map for correction of readout distortions in both T1w and T2w images. In addition, 10 min of eyes-open resting-state BOLD fMRI were collected using a multiband acquisition (TR = 800 ms, TE = 37 ms, multiband factor = 8, flip angle = 52°, FOV = 208 × 208 mm, voxel size = 2 mm^3^, 72 slices).

MRI data were processed using the HCP-MMP pipelines (see Glasser et al., 2013), with a full description of procedures provided in a recent report from our laboratory (Curtis et al., 2023). This process produced two main outputs: (1) each participant’s native-space cortical surface, generated by extracting white and pial surfaces from the T1w images and refined using T2w data (Dale et al., 1999); and (2) HCP-MMP parcellation files mapped and sampled to the individual cortical surface. Both outputs were imported into Brainstorm software (Tadel et al., 2011) and co-registered with MEG source data, allowing the identification of functionally meaningful auditory cortical regions in each participant’s native MRI space, which were subsequently used to label MEG source locations.

### 2.4 EEG MEG acquisition and processing

EEG and MEG acquisition and preprocessing followed procedures reported in our previous publication (López-Caballero et al., 2025). Briefly, combined EEG and MEG recordings were conducted in a magnetically shielded room (Imedco AG, Hägendorf, Switzerland) using a Megin Truix Neo system. EEG was recorded from 60 scalp electrodes mounted on a low-impedance cap (BrainCap MEG, Brain Vision LLC, Morrisville, NC, USA) according to the 10–10 system, with impedances kept below 10 kΩ. MEG was recorded using a 306-channel whole-head system (MEGIN Triux, Espoo, Finland) comprising 102 sensor triplets (1 magnetometer, 2 planar gradiometers). Data were sampled at 1000 Hz with an online 0.1–330 Hz bandpass filter. Concurrent EOG and ECG recordings were acquired to detect ocular and cardiac artifacts. The right mastoid served as reference, with the left mastoid recorded for later rereferencing. Head position relative to MEG sensors was continuously tracked during MEG recording via five HPI coils affixed to the EEG cap and registered to fiducial points (nasion and preauricular points) using a 3D digitizer (ISOTRAK; Polhemus, Inc., Colchester, VT) prior to MEG recording.

MEG data were denoised using the temporal extension of Signal Space Separation (tSSS; Taulu et al., 2004; Taulu and Simola, 2006), motion-corrected, and bad channels interpolated with MaxFilter (Elekta Neuromag). Using EEGLAB toolbox (Delorme and Makeig, 2004), EEG/MEG data were high-pass filtered at 0.5 Hz (12 dB/oct), noisy EEG sensors were interpolated, and data segments with large artifacts were rejected. At most, 1 VEOG, 1 HEOG, and 2 ECG artifacts were removed using AMICA in each participant. Next, using Brainstorm software (Tadel et al., 2011), data were low-pass filtered at 20 Hz (24 dB/oct) and EEG referenced to average mastoids. Epochs of −100 to 1,100 ms relative to stimulus onset were extracted and baseline corrected (using the pre-stimulus interval −100 to 0 ms). Epochs exceeding ±2.5 pT (MEG) or ±50 μV (EEG) were discarded. Remaining epochs were averaged separately for EEG and MEG.

On average, 84% of EEG epochs (SD = 19%) and 98% of MEG epochs (SD = 3%) survived artifact rejection. Two participants with less than 50% surviving EEG epochs were excluded, resulting in a final EEG sample of 30 participants; all 32 participants were retained for MEG analyses.

### 2.5 MEG source localization

We used cortical source modeling procedures as described in López-Caballero et al. (2025), with minor modifications. Dipolar sources were constrained to each participant’s native cortical surface from MRI processing and tessellated into an icosahedron mesh with 37,500 vertices per hemisphere. MEG sensors were co-registered to the structural images in two steps: (1) aligning digitized fiducial points (nasion, left and right pre-auricular points) with corresponding MRI landmarks, and (2) manually verifying registration using additional facial/head points and the MRI-derived head surface from Brainstorm Software (Tadel et al., 2011). The forward model employed overlapping spheres per sensor, and the noise covariance matrix was calculated from the −100 to 0 ms pre-stimulus baseline across all trials used for stimulus averages.

Cortical source activity was estimated separately for standard and deviant averages using minimum norm estimation (Gramfort et al., 2014). A linear inverse operator was constructed with a loose orientation constraint of 0.4 and depth weighting (Lin et al., 2006). Current density values at all 75,000 vertices were normalized using dynamic statistical parametric maps (dSPM) based on baseline variance. dSPM values were then averaged within each HCP-MMP parcel (A1 or PBelt) for statistical analyses, and source activity was also morphed to MNI-ICBM152 space with 10 mm smoothing for group-level visualization.

### 2.6 MMN calculation

Dual-rule MMN was calculated by subtracting the response to standards (Tone B in A-B pairs) from the response to deviants (repeated Tone A in A-A pairs). This approach was chosen instead of a direct comparison between Tone A standard and Tone A deviant considering stimulus-specific adaptation (SSA): in the Gap condition, the first Tone A in either A-B or A-A pairs is preceded by a 750 ms SOA, allowing more recovery from adaptation than the shorter 330 ms SOA preceding Tone B in an A-B pairs (Figure 1). Without this approach, responses to the deviant Tone A could appear larger due to reduced adaptation. Although adaptation is frequency-specific, numerous studies in rodents (Farley et al., 2010; Nieto-Diego and Malmierca, 2016; Taaseh et al., 2011) and humans (Herrmann et al., 2013, 2015) indicate that it is not confined to the repeated frequency but generalizes across nearby frequencies over a range of several hundred Hz. Herrmann et al. (2013) supported that the Gaussian spread of adaptation along the tonotopic axis depends on spectral variance and can extend up to ~800 Hz away from the standard tone within the 700–2,500 Hz range. Herrmann et al. (2015) further showed that co-adaptation functions centered at 1 kHz similarly extended across a frequency range up to ~1930 Hz, with the width modulated by statistical context. Thus, for the 200 Hz frequency difference used here (1 kHz vs. 1.2 kHz), responses to repeated Tone A and to Tone B are expected to be similarly adapted. For the small frequency difference used here (1 kHz vs. 1.2 kHz), responses to Tone B and repeated Tone A are therefore expected to be similarly adapted.

### 2.7 Statistical analysis

**Figure 2:**
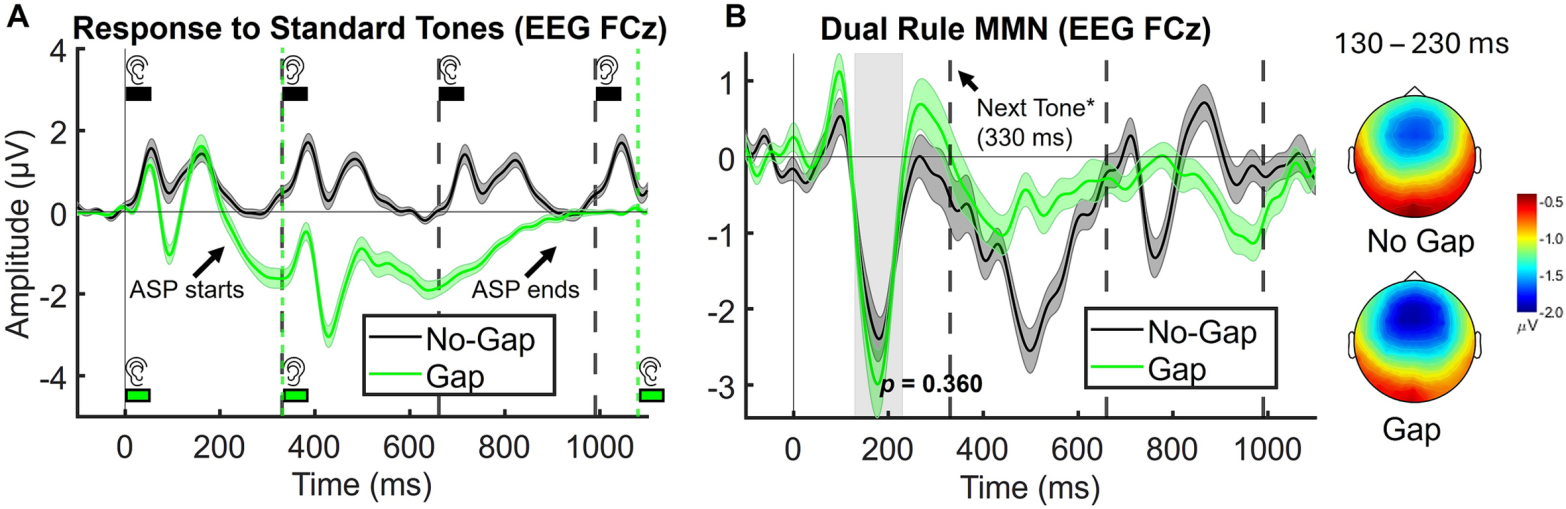
Grand-average waveforms (*n* = 30) at FCz (EEG). **(A)** Illustration of the Auditory Segmentation Potential (ASP) in the Gap condition, elicited by standard tones in the dual-rule paradigm. Black squares indicate tone sequences in the No-Gap condition, and green squares indicate sequences in the Gap condition, where a 750-ms SOA was inserted between tone pairs. Tones alternated in spatial location and in frequency (1 kHz vs. 1.2 kHz, not depicted). **(B)** Dual-rule MMN derived by subtracting responses to standards (Tone B) from deviants (repeated Tone A). The shaded gray rectangle marks the analysis window (130–230 ms). Corresponding scalp topographies for the dual-rule MMN are shown for each condition. Shaded areas indicate the standard error of the mean (SEM). * Arrow marks the next tone in the sequence, which occurs only in the No-Gap condition.

For EEG analyses, MMN amplitude was quantified at the FCz electrode as the mean amplitude over a 130–230 ms time window, corresponding to the peak observed in the grand-average waveform (Figure 2B). Paired-samples t-tests were then conducted to compare this MMN amplitude between the Gap and No-Gap conditions.

**Figure 3:**
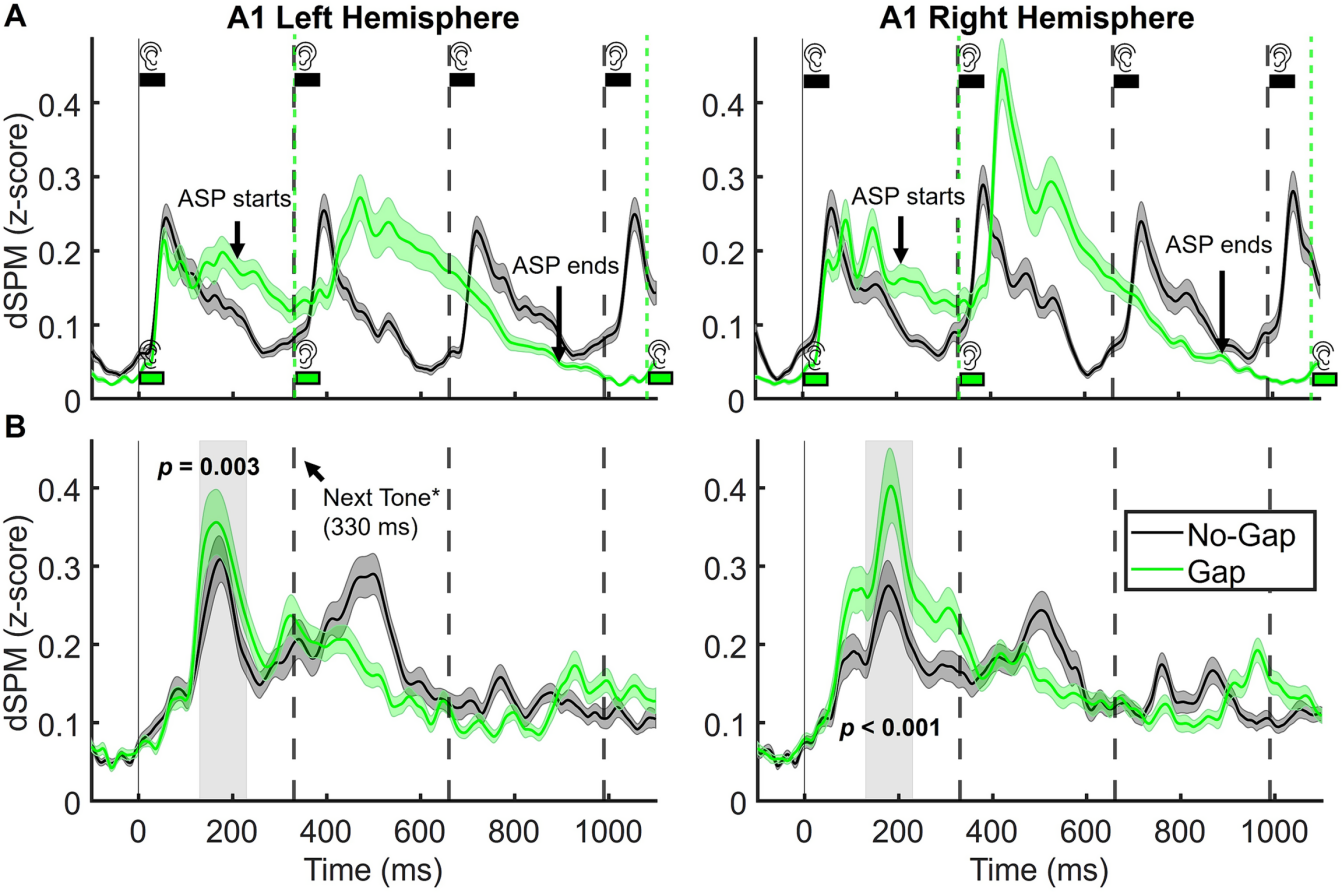
**(A)** Grand-average source time-courses (dSPM values, Left and Right PBelt) elicited by standard tones in the dual-rule paradigm, illustrating the Auditory Segmentation Potential (ASP) in the Gap condition in the source domain. **(B)** Grand-average MEG MMN source time-courses (dSPM values) from A1, as defined by the HCP-MMP parcellation, shown separately for the left (left panel) and right (right panel) hemispheres. The shaded gray rectangle marks the analysis window (130–230 ms). Shaded areas indicate the standard error of the mean (SEM). * Arrow marks the next tone in the sequence, which occurs only in the No-Gap condition.

For MEG analyses, repeated-measures ANOVAs were performed with two within-subject factors: Condition (Gap vs. No-Gap) and Hemisphere (Left vs. Right). MMN peak amplitudes were averaged over the 130–230 ms time window and extracted from dSPM values at the A1 source, with the time window selected based on the MMN peaks in the grand-average waveform (Figure 3). Exploratory pairwise comparisons, Bonferroni-corrected for multiple comparisons, were conducted to examine Condition effects separately within each hemisphere. Effect sizes were reported using partial eta-squared for ANOVAs and Cohen’s d for t-tests. Whenever the assumption of sphericity was violated, degrees of freedom were adjusted using Greenhouse–Geisser corrections. All statistical analyses were carried out using IBM SPSS Statistics (Version 26).

## 3 Results

### 3.1 EEG sensor data

Grand-average responses to standard predictable tones at FCz electrode are provided in Figure 2A. In the No-Gap condition, sizable N100 responses to each of the standard tones were observed (Figure 2A, black trace), peaking at approximately 90 ms post-stimulation. In addition, an auditory segmentation potential (ASP) was evident in the Gap condition (Figure 2A, green trace), manifesting as a sustained negative-going potential starting around 200 ms after the first tone of the pair and returning to baseline just prior to the onset of the next pair (approximately 1,080 ms, indicated by the green vertical dotted line), consistent with previous observations (Coffman et al., 2016). Individual N100 responses to the first and second tones of the pair were also apparent in the Gap condition.

**Figure 4:**
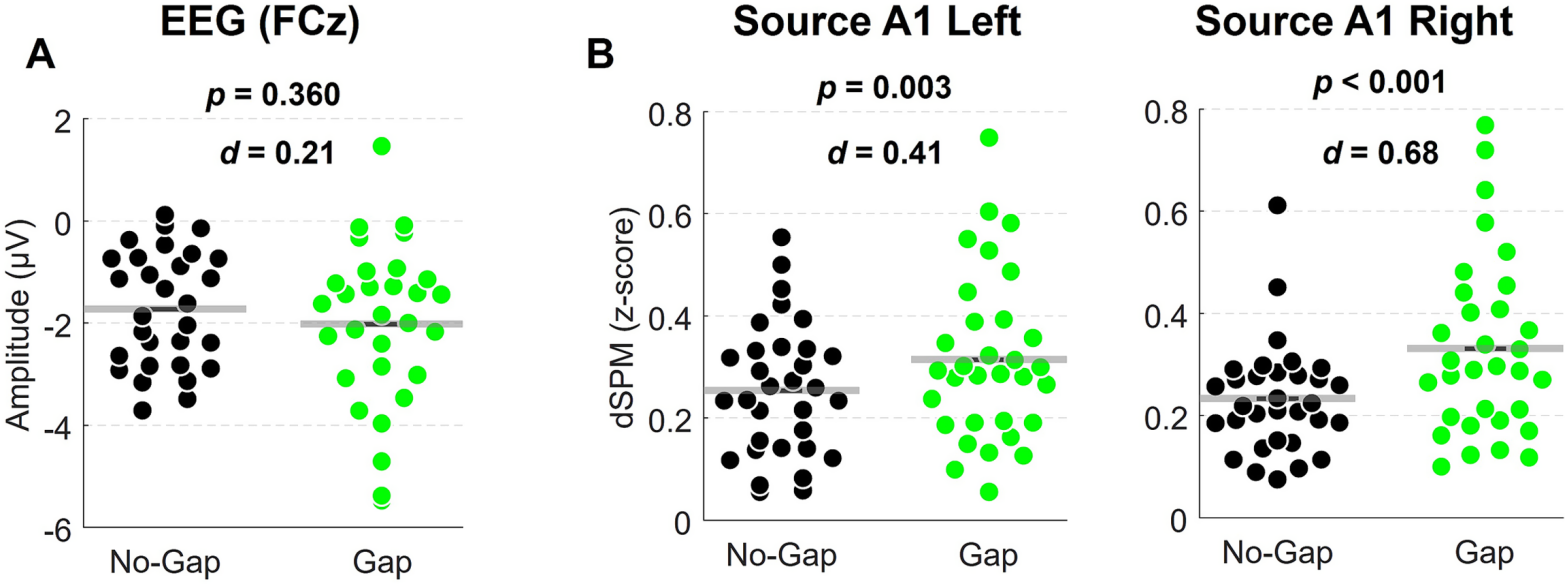
Scatter plots of dual-rule MMN mean amplitudes at EEG FCz **(A)** and at left and right A1 MEG sources **(B)**. For each plot, *p* values from paired-samples t-tests (EEG) or Bonferroni-corrected pairwise comparisons (MEG) are shown, along with Cohen’s *d* effect sizes. Significant differences between No-Gap and Gap conditions were observed only for A1 MEG sources.

Difference waves obtained by subtracting responses to standards (Tone B) from responses to deviants (repeated Tone A) revealed a clear MMN peaking at approximately 180 ms post-stimulation, with the expected frontocentral scalp distribution (Figure 2B). Mean MMN amplitudes (130–230 ms) at FCz were numerically larger in the Gap condition (M = −2.01 μV, SD = 0.29) compared to the No-Gap condition (M = −1.72 μV, SD = 0.20), although this difference did not reach statistical significance (t_(29)_ = 0.93, *p* = 0.36, *d* = 0.17, see Figure 4A). In addition, a later negative deflection was observed in the No-Gap condition at approximately 500 ms (Figure 2B). This effect will be considered further in the Discussion.

### 3.2 MEG sources data

**Figure 5:**
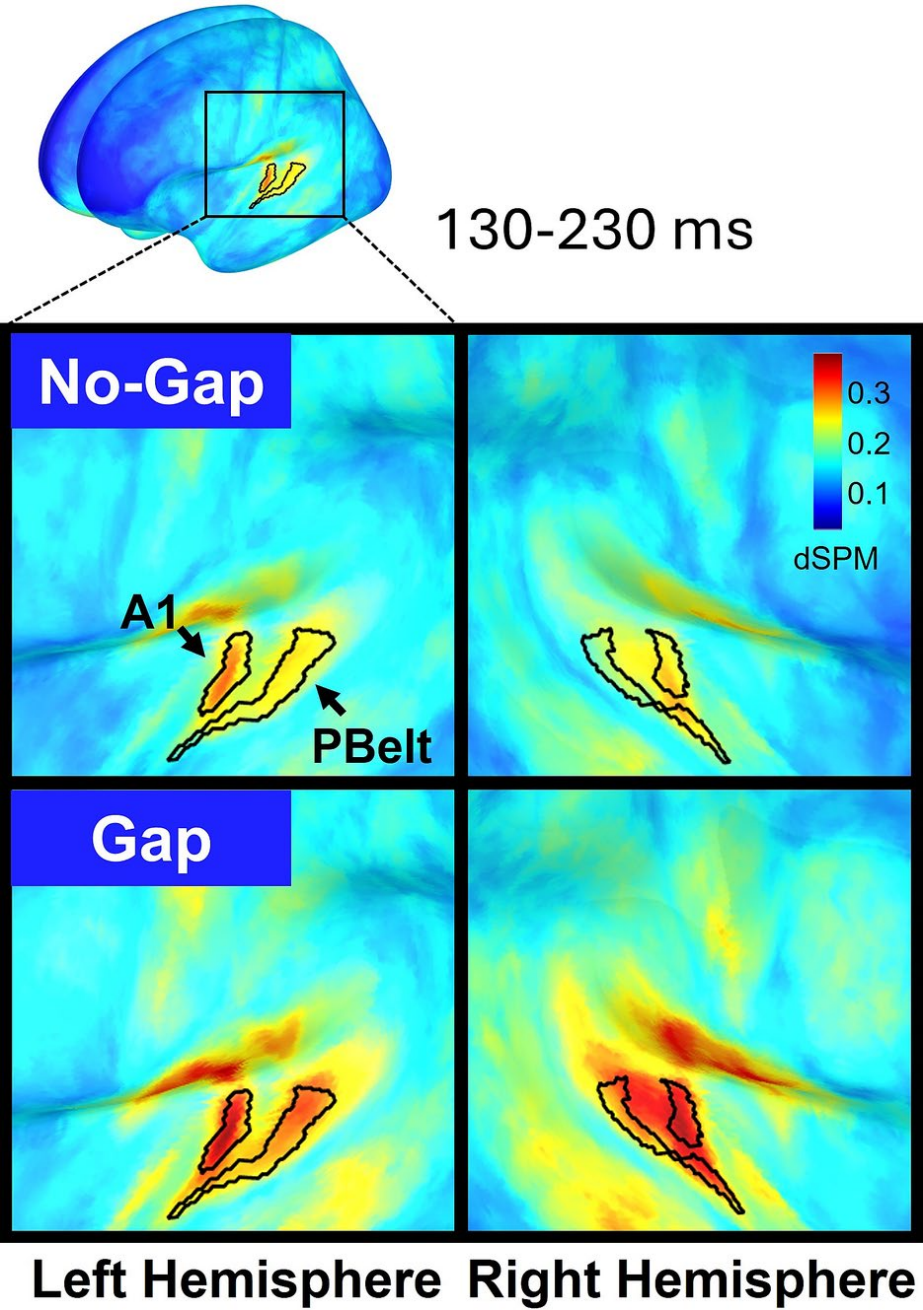
Cortical source maps of the dual-rule MMN response projected onto the MNI-ICBM152 brain template and averaged across participants (*n* = 32). Activity is displayed separately for the left (left panels) and right (right panels) hemispheres, with No-Gap (top) and Gap (bottom) conditions shown. MMN dSPM values were averaged over the 130–230 ms time window. Within the auditory cortex, the regions showing the strongest responses (primary auditory cortex -A1- and parabelt -PBelt-) are delineated according to the Human Connectome Project multimodal parcellation (HCP-MMP).

Source reconstruction of MEG data revealed that MMN activity was primarily localized to bilateral primary auditory cortex (A1) and parabelt (PBelt) areas, appearing as two spatially distinct clusters of activation (Figure 5). Given A1 exhibited higher dSPM values, our main analyses focused on this region. However, parallel analyses in Pbelt areas yielded identical statistical effects, and these results are provided in the Supplementary materials.

Figure 3A shows source-level waveforms in bilateral A1 elicited by standard stimuli in both experimental conditions. Visual inspection of the waveforms suggests that individual evoked responses to each tone are present in the No-Gap condition. In the Gap condition, responses to individual tones overlapped with a slow-wave potential extending across the gap between tone pairs (approximately 200–1,080 ms, as also seen in the EEG), likely reflecting ASP in the source domain (not previously reported). This response also appeared more pronounced over the right hemisphere, although statistical tests were conducted only for MMN.

Figure 3B shows MMN waveforms in bilateral A1, plotted separately for each hemisphere. Robust dual-rule MMN responses were observed bilaterally, with clear peaks at approximately 180 ms. In addition, a later negative deflection emerged in the No-Gap condition around 500 ms, consistent with the pattern observed at the EEG level.

The MMN source response in A1 was larger in the Gap condition relative to No-Gap (*F*_(1,31)_ = 26.07, *p* < 0.001, $\eta_{p}^{2}$ = 0.457), with no differences between hemispheres (F_(1,31)_ = 0.007, *p* = 0.935, $\eta_{p}^{2}$ = 0.000) and no significant Condition × Hemisphere interaction (F_(1,31)_ = 2.07, *p* = 0.160, $\eta_{p}^{2}$ = 0.063), indicating that the effect of Condition was similar in left and right A1. For completeness, exploratory pairwise comparisons within each hemisphere confirmed that the Gap condition elicited stronger MMN responses in both left (Mean difference = −0.061, 95% CI [−0.099, −0.023], *p* = 0.003, *d* = 0.41) and right A1 (Mean difference = −0.098, 95% CI [−0.14, −0.054], *p* < 0.001, *d* = 0.68). Mean dual-rule MMN values extracted from each participant at A1 are displayed in Figure 4B for each hemisphere.

## 4 Discussion

In the present study, we examined how auditory segmentation shapes deviance detection in a complex dual-rule MMN paradigm. By comparing continuous and temporally segmented conditions, we found that inserting a 750 ms gap between tone pairs elicited a robust auditory segmentation potential and led to significantly larger MMN responses in bilateral auditory cortex, as measured with MEG. This enhancement was not observed in scalp EEG. The findings indicate that temporal segmentation facilitates the perceptual grouping of tone pairs into auditory objects, enabling predictions at both the level of individual tones and the pair structure itself.

### 4.1 Segmentation and hierarchical prediction errors

A central implication of our findings is that segmentation facilitates the auditory system to organize sequential sounds into perceptual objects, in this case tone pairs, and to generate predictions at multiple nested levels. When stimuli are temporally continuous, as in the No-Gap condition, predictions may be largely constrained to local feature regularities. By contrast, the Gap condition facilitates tones to be grouped into discrete perceptual units, supporting predictions about both the features of the upcoming sound and the integrity of the object-level pair. Deviants that break these predictions simultaneously disrupt expectations at multiple levels: the immediate expectation of a right-biased high tone following a left-biased low tone, and the broader expectation of coherence at the object level, namely that the pair consists of A-B rather than A-A. Both at EEG and MEG levels (Figures 2A, 3A), distinctive evoked responses are elicited by sound patterns where a 750 ms SOA intervenes between tone pairs vs. a constant 330 ms condition (see black vs. green sound sequences represented): an ongoing lower-frequency component emerges between the first tone pair and expands until the onset of the next pair. This suggests an underlying neural process that encodes the entire tone pair as a coherent perceptual unit, likely reflecting the joint action of auditory-object boundary identification and subsequent auditory segmentation. In turn, this underlying segmentation process could shape the formation and maintenance of predictive models at the object level (tone-pair), thus enhancing sensitivity to violations of both local feature regularities and global object structure. This is consistent with the observed MMN amplitude increase for the Gap condition, which reflects not only the detection of feature deviants but also the disruption of predictions relying in object-level representations.

This interpretation aligns with prior demonstrations that MMN amplitudes increase when regularities are defined across extended sequences rather than single sounds. Wacongne et al. (2011) showed that prediction errors are larger when omissions violate both local expectations and higher-level global patterns, indicating multi-level predictive processing. Similarly, List et al. (2007) reported that global pattern violations elicit mismatch responses even in the absence of local irregularities, highlighting the priority of higher-order structure. Our findings extend these observations by showing that temporal segmentation can enforce such higher-order structure, enabling nested representations to emerge.

### 4.2 ASP as a potential contributor to MMN enhancement

An alternative but not mutually exclusive explanation is that the ASP may contribute to the MMN increase. We observed ASP as a sustained negative-going potential in the Gap condition, beginning approximately 200 ms after the onset of the first tone of the pair and returning to baseline just prior to the onset of the next pair (~1,080 ms; Figure 2A). This prolonged temporal window overlaps with the MMN time window, raising the possibility of interaction. A purely additive effect is unlikely, as any component equally engaged by standards and deviants would largely cancel in the difference waveform. Conversely, if the ASP is differentially modulated (e.g., larger or more prolonged for deviants) then this asymmetry could enhance the deviant-minus-standard difference, amplifying the measured MMN amplitude. In this scenario, the observed enhancement would reflect an interaction between segmentation-related activity and deviance detection, rather than a simple coexistence of independent components. Such a mechanism would parallel findings for the object-related negativity (ORN), which overlaps temporally with the N1-P2 complex but is considered a distinct marker of concurrent object segregation (Alain, 2007).

Several features of the ASP may be generalizable across paradigms. Temporally, it spans the entire interval between the first tone of a pair and the onset of the subsequent pair, potentially reflecting hierarchical segmentation at the level of grouped tones. In EEG, this sustained negativity is clearly visible at frontocentral electrodes and may reflect contributions from multiple overlapping cortical sources. In MEG, the response is more focal and source-localized, typically in bilateral A1, and thus may capture only a subset of the distributed activity seen in EEG. A useful marker for future studies could be the temporal boundaries of the ASP: whether the negativity always starts between the first and second tone of the first pair and ends before the first tone of the next pair, how it behaves for longer SOAs (>750 ms), or when larger groupings are used (e.g., triplets instead of tone pairs). Further characterization could include analyses of frequency components, as the ASP appears slower than the brief, time-locked MMN peak.

Although examining the relationship between ASP and MMN was not a primary aim of the present study, future work could be designed to explicitly test this interaction. For example, grouping salience could be manipulated independently of deviance (e.g., by varying the duration or number of tones within a group, or by parametrically increasing the inter-group SOA), allowing a systematic assessment of how segmentation strength modulates MMN. In addition, deviance could be introduced at different hierarchical levels (e.g., within pairs by altering tone identity or between pairs by keeping the A-B pair intact but occasionally omitting an entire pair or changing its order), enabling the dissociation of ASP-related activity from deviance-specific responses through carefully defined analysis windows. Longer gap durations or multi-tone groupings would also make it possible to examine whether the sustained ASP negativity scales with group structure or inter-group SOA, providing a more direct test of its contribution to MMN enhancement.

### 4.3 Difference between EEG and MEG source-level findings

Our MEG source estimates were consistent with MMN generators in bilateral auditory cortex reported in EEG/MEG source reconstruction studies (Alho, 1995; Giard et al., 1990; Hari et al., 1992; Rinne et al., 2000) and with fMRI studies (Molholm et al., 2005). Furthermore, given the complex nature of our paradigm (as compared with simple feature MMN deviations), our source estimates also align with studies showing that more complex regularities recruit secondary auditory areas (Salisbury, 2012; Schröger et al., 2007), in our case, the parabelt. However, we did not observe frontal sources that have been implicated in higher-order deviance processing (Bekinschtein et al., 2009; Bonetti et al., 2022). One potential explanation for this is that MEG is relatively less sensitive to radially oriented sources, such as those in the frontal gyri, and to deep anterior regions (Baillet, 2017; Hillebrand and Barnes, 2002; Marinkovic et al., 2004).

On the other hand, our source-localized MEG results revealed enhanced MMN responses in the Gap condition, while EEG scalp responses at FCz did not differ significantly between conditions. This suggests that the effect of segmentation is most clearly expressed at the source level. The reason behind this finding is likely multifactorial. One possible reason is that segmentation may preferentially enhance activity in tangentially oriented temporal sources, which are detected more effectively by MEG than by scalp EEG (Baillet, 2017; Hillebrand and Barnes, 2002; Marinkovic et al., 2004). Alternatively, if segmentation primarily boosts the temporal (A1) contribution without proportionally affecting frontal MMN sources, the scalp topography may change little even as the temporal source grows. In turn, temporal sources alone may not produce a large enough change in scalp topography to yield significant amplitude differences at FCz, particularly if frontal contributions are weak or absent (Giard et al., 1990; Rinne et al., 2000). Methodological factors may also contribute to our results. The 130–230 ms time window used for MMN extraction encompasses not only the negative MMN peak but also the onset of positive components such as the P3a (Polich, 2007), which can partially mask condition differences at the scalp. By contrast, the dSPM z-scores used for MEG source analysis are unsigned and more robust to polarity reversals. Taken together, these considerations suggest that the absence of EEG effects reflects the lower sensitivity of scalp measurements rather than a true absence of underlying neural modulation.

### 4.4 The later negativity in the No-Gap condition

In addition to the first MMN peak at ~180 ms where we focused our analyses, a later negative deflection was observed in the No-Gap condition at approximately 500 ms (Figure 2B). In this condition, both standards (Tone B) and deviants (repeated Tone A) are followed by a subsequent Tone A after 330 ms (Figure 1). Accordingly, the later negativity reflects the subtraction of responses to a Tone A following a repeated Tone A from those to a Tone A following a Tone B. The presence of this MMN-like response suggests that a Tone A occurring after a repeated Tone A may itself be processed as a deviant in this No-Gap condition. Given the continuous stimulation (constant 330 ms SOA), a third consecutive Tone A in the sequence (…A-B, A-B, A-A, A-B…) could plausibly violate the expectation that Tone A is consistently followed by Tone B, thus eliciting a secondary mismatch response. While the exact mechanism underlying this late negativity remains uncertain, it may reflect either a secondary mismatch response to this higher-order sequence violation by the third tone A, or a secondary deviance process related to the processing of the first deviant tone. To disentangle these possibilities, future work could systematically manipulate the sequence structure, for example by introducing occasional sequences in which a third consecutive Tone A is either omitted or replaced with a Tone B, directly testing whether the late negativity reflects a secondary deviance response to A-B pattern violations.

### 4.5 Relation to auditory scene analysis and prior grouping studies

Our results also contribute to the broader literature on auditory scene analysis, which highlights the role of perceptual organization in deviance detection. A number of studies have shown that MMN amplitude depends not only on simple feature irregularities but also on whether the brain has organized sounds into coherent perceptual streams (Hikita et al., 2020; Sussman, 2005; Sussman-Fort and Sussman, 2014; Suzutani et al., 2025). In paradigms where perceptual grouping is facilitated, such as by minimizing frequency overlaps, MMN responses are typically stronger, reflecting the brain’s sensitivity to deviations from organized perceptual structures. For example, Smith and Joshi (2014) observed that conditions facilitating perceptual grouping of target tones not only improved N1 timing but also produced additional sustained negativities alongside MMN, suggestive of grouping-related processes. Our findings parallel these in showing that segmentation, by facilitating temporal grouping, similarly increases MMN amplitudes.

Importantly, however, the present paradigm is distinct from traditional stream segregation studies. Whereas segregation involves distinguishing simultaneous or interleaved streams based on pitch or location, segmentation refers to the temporal chunking of sequential sounds into units, often facilitated by gaps. The tones in our paradigm were too close in frequency (1 kHz vs. 1.2 kHz) to yield robust stream segregation based on frequency (Hikita et al., 2020; Suzutani et al., 2025). Thus, the observed enhancements cannot be explained by segregation of independent streams. Instead, segmentation uniquely accounts for our findings, showing that temporal grouping can serve as a complementary mechanism to segregation in organizing complex auditory input. Specifically, classic segregation studies demonstrate that perceptual organization influences not only the magnitude but also the structure of deviance representations indexed by MMN. Sussman (2005) showed that once streams are segregated, stable contextual structure determines whether closely-timed successive deviants are represented as one integrated event or as two distinct events. Our temporal segmentation may play an analogous role by introducing clear temporal boundaries that stabilize representations at the level of tone pairs, resulting in enhanced predictive precision. Further, while segregation requires a buildup period before MMN responses reach their maximal expression (Sussman-Fort and Sussman, 2014), segmentation provides an immediate organizational cue. Finally, while segregation supports parallel predictive models across streams, segmentation creates hierarchical predictive structure within a stream, allowing local and global regularities to be encoded simultaneously. Together, these parallels suggest that temporal segmentation and stream segregation rely on different acoustic cues but converge functionally to enhance predictive processing in complex auditory scenes.

Our results further align with prior work on temporal segmentation, highlighting how specific temporal windows structure auditory representations. Atienza et al. (2003) showed that tones presented in rapid succession were integrated into larger perceptual units within a temporal window of approximately 240 ms, such that MMN was elicited only when inter-train intervals remained within this limit. Similarly, Sussman and Gumenyuk (2005) demonstrated that five-tone sequences were grouped in memory only at sufficiently short onset-to-onset intervals (200 ms), suggesting the critical role of temporal proximity in establishing predictive representations. While these studies focused on integration within a single perceptual unit (tones within a train or sequence) our study extends these findings by examining hierarchical segmentation across two temporal scales. The 330 ms within-pair SOA allows the auditory system to bind individual tones into a pair-level unit, and the 750 ms gap between pairs provides a clear temporal boundary that supports higher-order predictive encoding.

### 4.6 Limitations and future directions

Several limitations warrant consideration. First, the interpretation of the late negativity in the No-Gap condition remains speculative and requires targeted experiments to determine whether it reflects a true mismatch response or a byproduct of overlapping sequence responses. Second, stimulus-specific adaptation (SSA) may have influenced our results. We attempted to minimize this by comparing responses to Tone B with those to repeated Tone A, reasoning that both should be similarly adapted given their small frequency difference (Herrmann et al., 2013, 2015). However, if SSA were strictly frequency-specific (such that Tone B did not undergo adaptation from a preceding Tone A) then our subtraction approach could still yield residual differences not entirely attributable to deviance detection. Moreover, while MEG provided clear evidence for enhanced MMN in the Gap condition, the absence of EEG effects may reflect methodological limitations, and complementary approaches (e.g., intracranial recordings) would be valuable.

Another potential limitation concerns our experimental design, where MMN is calculated by comparing the response to Tone B in the A-B standard with the response to the second Tone A in the A-A deviant. Tone A and Tone B differ in frequency (1 kHz vs. 1.2 kHz) and laterality bias (right vs. left ear). Therefore, part of the observed response difference could reflect simple sensory-evoked differences rather than a genuine prediction-error or deviance-detection process. While we cannot entirely rule out this possibility, the tones were close enough in frequency that they are unlikely to induce robust stream segregation (Hikita et al., 2020; Suzutani et al., 2025). In addition, both tones were presented frequently across the experiment, so any basic physical difference would be expected to influence Gap and No-Gap conditions similarly.

While this study focused on evoked responses, future work could apply time-frequency analyses to investigate oscillatory mechanisms of auditory segmentation. Teng et al. (2018) showed that theta-band activity supports auditory segmentation, or “chunking,” of continuous streams into perceptual units. Given that MMN generation relies strongly on theta-band activity (e.g., Javitt et al., 2018), such analyses could reveal whether the Gap condition enhances theta synchronization or phase coherence, reflecting stronger segmentation and predictive coding.

Future studies could further address the relationship between temporal segmentation and MMN amplitude by systematically manipulating temporal parameters such as SOA, gap duration, and tone-group structure. Such work could assess whether segmentation effects generalize across different types of auditory regularities. For instance, the precise temporal thresholds for perceptual object formation in tone-pair paradigms remain to be delineated. Parametric variation of the SOA between perceptual objects (e.g., 500, 750, 1,000 ms) would allow testing of the optimal temporal window for segmentation-based object formation and its impact on deviance detection.

## 5 Conclusion

In sum, our results demonstrate that auditory segmentation amplifies prediction error responses in a complex dual-rule paradigm. By temporally grouping tones into discrete pairs, segmentation enables the auditory system to establish predictive models at both tone-level and object-level scales, such that deviants violate multiple nested expectations. The associated ASP may further contribute to the enhanced MMN by selectively overlapping with deviant responses. These findings bridge research on auditory scene analysis, predictive coding, and electrophysiological correlates of segmentation, highlighting the importance of temporal grouping as a mechanism that strengthens deviance detection.

## Data Availability

The data that support the findings of this study are available from the corresponding author upon reasonable request.
