# Supplementary material for: Auditory object segmentation amplifies prediction error signals in a complex MMN paradigm
Source: Front Neurosci. 2025 Dec 5;19:1695952. doi: 10.3389/fnins.2025.1695952 (PMC12715717; doi:10.3389/fnins.2025.1695952)
Supplement: Supplementary file 1 [file Data_Sheet_1.docx]

**Supplementary Materials**

**Summary of results at PBelt**: The MMN source response in A1 was larger in the Gap condition relative to No-Gap (F_(1,31)_ = 21.26, *p* < .001, $ƞ_{p}^{2}$ = .407), with no differences between hemispheres (F_(1,31)_ = 0.015, *p* = .902, $ƞ_{p}^{2}$ = .000) and no significant Condition × Hemisphere interaction (F_(1,31)_ = 2.01, *p* = .165, $ƞ_{p}^{2}$ = .061), indicating that the effect of Condition was similar in left and right A1. For completeness, exploratory pairwise comparisons within each hemisphere confirmed that the Gap condition elicited stronger MMN responses in both left (Mean difference = -0.052, 95% CI [-0.085, -0.020], *p* = .002, *d* = .41) and right A1 (Mean difference = -0.078, 95% CI [-0.11, -0.042], *p* < .001, *d* = .67).


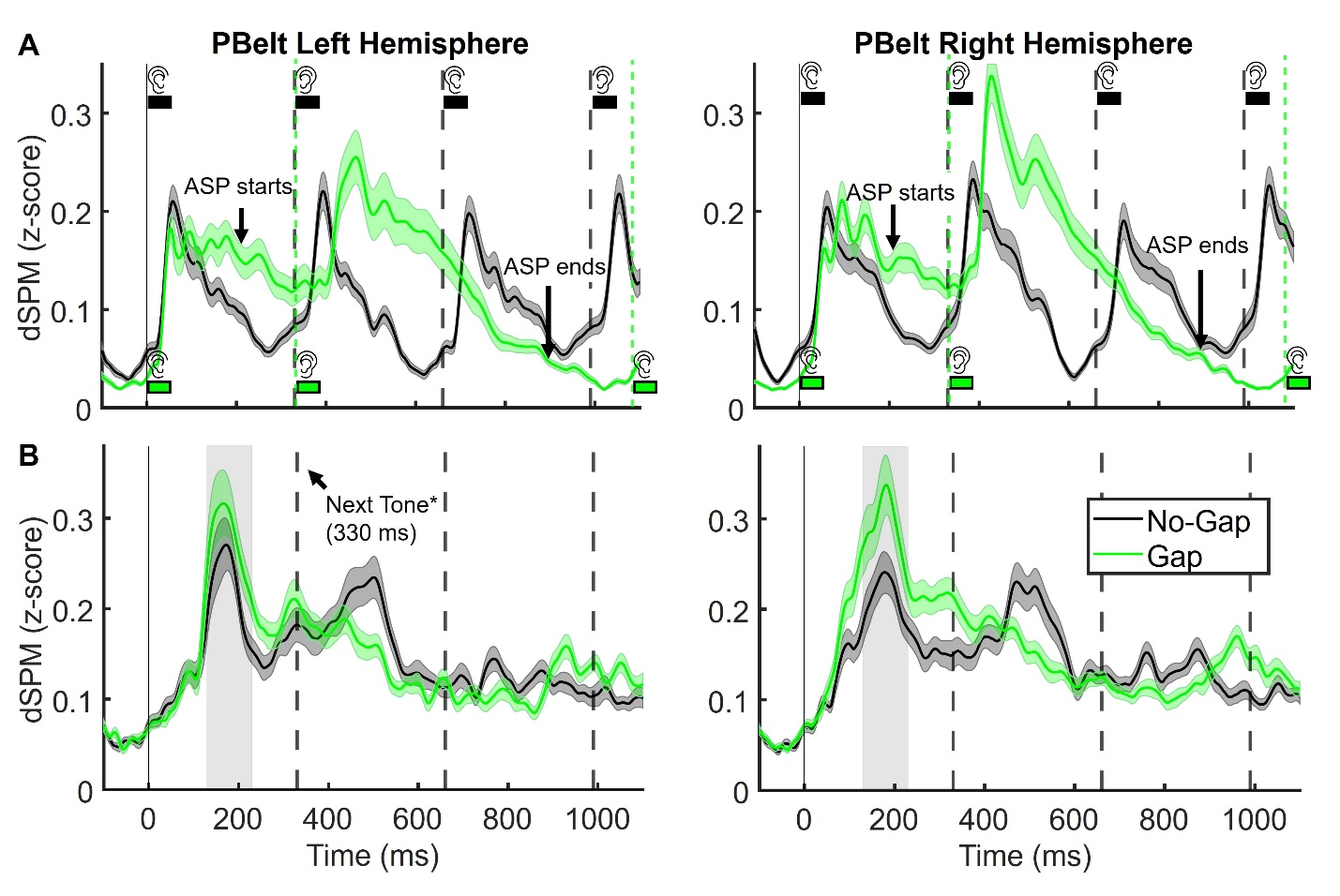


Supplementary Figure 2. (A) Grand-average source time-courses (dSPM values, Left and Right PBelt) elicited by standard tones in the dual-rule paradigm, illustrating the Auditory Segmentation Potential (ASP) in the Gap condition in the source domain. (B) Grand-average MEG MMN source time-courses (dSPM values) from PBelt, as defined by the HCP-MMP parcellation, shown separately for the left (left panel) and right (right panel) hemispheres. The shaded gray rectangle marks the analysis window (130-230 ms). Shaded areas indicate the standard error of the mean (SEM). * Arrow marks the next tone in the sequence, which occurs only in the No-Gap condition.


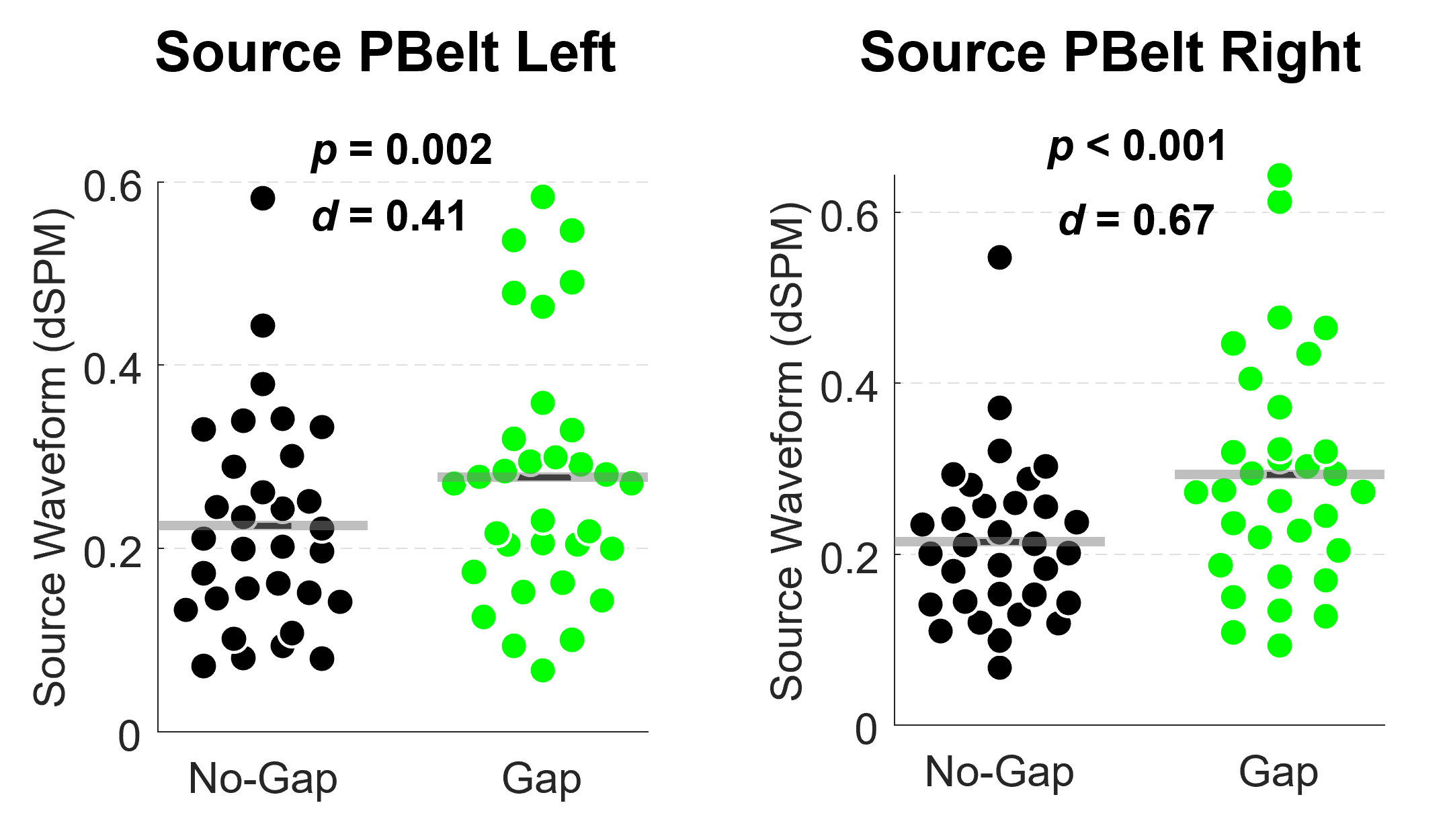


Supplementary Figure 3. Scatter plots of dual-rule MMN mean amplitudes at left and right PBelt MEG sources. For each plot, *p* values from Bonferroni-corrected pairwise comparisons are shown, along with Cohen’s *d* effect sizes. Significant differences between No-Gap and Gap conditions were observed for both left and right PBelt MEG sources.
